# Diagnostic and Prognostic Value of Conventional Brain MRI in the Clinical Work-Up of Patients with Amyotrophic Lateral Sclerosis

**DOI:** 10.3390/jcm9082538

**Published:** 2020-08-06

**Authors:** Giovanni Rizzo, Anna Federica Marliani, Stella Battaglia, Luca Albini Riccioli, Silvia De Pasqua, Veria Vacchiano, Rossella Infante, Patrizia Avoni, Vincenzo Donadio, Massimiliano Passaretti, Ilaria Bartolomei, Fabrizio Salvi, Rocco Liguori, on behalf of the BoReALS group

**Affiliations:** 1 Department of Biomedical and Neuromotor Sciences, University of Bologna, Via Altura 3, 40139 Bologna, Italy; silvia.depasqua@hotmail.it (S.D.P.); veriavacchiano@gmail.com (V.V.); rossellainfante5@gmail.com (R.I.); patrizia.avoni@unibo.it (P.A.); massimili.passaretti@studio.unibo.it (M.P.); rocco.liguori@unibo.it (R.L.); 2 IRCCS Istituto delle Scienze Neurologiche di Bologna, Via Altura 3, 40139 Bologna, Italy; federica.marliani@gmail.com (A.F.M.); stella.battaglia@isnb.it (S.B.); luca.albiniriccioli@ausl.bologna.it (L.A.R.); vincenzo.donadio@unibo.it (V.D.); ilaria.bartolomei@gmail.com (I.B.); fabrizio.salvi@gmail.com (F.S.)

**Keywords:** ALS, MRI, MND, corticospinal tract, motor cortex, SWI, FLAIR

## Abstract

Clinical signs of upper motor neuron (UMN) involvement are important in the diagnosis of amyotrophic lateral sclerosis (ALS) though are often difficult to analyze. Many studies using both qualitative and quantitative evaluations have reported abnormal Magnetic Resonance Imaging (MRI) findings at the level of the pyramidal pathway in patients with ALS. Although the most interesting results were obtained by quantitative studies using advanced MR techniques, the qualitative evaluation of MRI images remains the most-used in clinical practice. We evaluated the diagnostic and prognostic contribution of conventional 3T-MRI in the clinical work-up of ALS patients. Two neuroradiologists retrospectively assessed 3T-MRI data of 93 ALS patients and 89 controls. The features of interest were corticospinal tract (CST) T2/FLAIR hyperintensity, motor cortex (MC) T2*/SWI hypointensity, and selective MC atrophy. All MRI features were significantly more prevalent in ALS patients than in controls. The simultaneous presence of CST FLAIR hyperintensity and MC SWI hypointensity was associated with the highest diagnostic accuracy (sensitivity: 70%; specificity: 81%; positive predictive value, PPV: 90%; negative predictive value, NPV: 51%; accuracy: 73%) and a shorter survival (HR: 6.56, *p* = 0.002). Conventional 3T-MRI can be a feasible tool to detect specific qualitative changes based on UMN involvement and to support clinical diagnosis of ALS. Importantly, CST FLAIR hyperintensity and MC SWI hypointensity are predictors of shorter survival in ALS patients.

## 1. Introduction

Amyotrophic lateral sclerosis (ALS), is a degenerative motor neuron disease, characterized by a progressive weakness of bulbar, limb, thoracic, and abdominal muscles [1].

The diagnosis of ALS is based on the detection of clinical signs of upper motor neuron (UMN) involvement, clinical and electrophysiological signs of lower motor neuron (LMN) impairment, and the exclusion of ALS mimics according to the revised El Escorial criteria [2].

While electromyography (EMG) can be used to detect signs of LMN impairment, signs related to UMN involvement can be difficult to detect since they can be masked by the signs of LMN impairment. This has led to a need to identify biomarkers that are sensitive and specific to UMNs. In terms of clinical presentation and survival, ALS is known to be extremely heterogeneous [3].

Conventional Magnetic Resonance Imaging (MRI) of the brain is routinely performed in the diagnostic workup for ALS in order to rule out other pathologies that present involvement of UMNs. However, over the last few decades, numerous studies using both qualitative and quantitative evaluation have reported abnormal MRI findings at the level of the pyramidal pathway in patients with ALS [4,5]. The most interesting results were obtained in studies using advanced MR techniques that are able to detect subtle changes in the motor cortex or the corticospinal tract (CST). However, these techniques are not yet ready for use in clinical practice. The qualitative evaluation of MRI images remains the most-used approach in the diagnostic procedure that is applied to these patients, and many studies report variations in definite radiological features. In patients with ALS, conventional MRI (i.e., T2-weighted, proton density (PD)-weighted, and fluid attenuated inversion recovery (FLAIR) sequence) can be used to detect changes in signal intensity along the CST [4,5]. A low-signal-intensity rim, probably related to iron deposition, can be found in T2-weighted images and, even more prominently, in T2*-weighted and susceptibility-weighted images (SWI) of the precentral cortex of ALS patients [4]. Furthermore, the motor cortex can be visibly atrophic [4,5]. Such features seem to be more easily detectable using high-field scanners.

The aim of this study was to evaluate the potential contribution of conventional 3T-MRI in the diagnosis and prognosis of a large sample of ALS patients.

## 2. Methods

### 2.1. Subjects

We retrospectively evaluated the MRI exams of 93 ALS patients satisfying the revised El Escorial criteria [2] (55 men and 38 females; age 62.8 ± 10.1, mean ± SD) and 89 controls (56 men and 33 females; age 60.2 ± 9.5, mean ± SD). The MRI exams were performed between 2009 and 2017 at a single center (Bellaria Hospital, Bologna, Italy). Seventy patients were longitudinally followed at our hospital. Patients underwent neurological, pneumological, physiatric and nutritional evaluations every three-four months, according to the clinical practice. The remaining 23 patients moved to other centers, after a variable observation time. However, we have tried to obtain as much follow-up information as possible, by contacting the reference doctors or family members. For the purpose of this study, we searched for information regarding the prognosis of ALS patients, i.e., the time to non-invasive ventilation (NIV)/tracheostomy, to parenteral nutrition/percutaneous endoscopic gastrostomy (PEG), and to death and the decline of revised ALS Functional Rating Scale (ALSFRS-R) score. Complete clinical follow-up information was available for 73 patients. The MRI data used in this study were obtained during the diagnostic work-up. Longitudinal MRI data were not available. The patients were stratified according to the clinical phenotype (classic, bulbar, prevalent UMN, and prevalent LMN, including flail-arm and flail-leg variants) based on clinical onset and predominant signs [6,7]. Twelve patients had mutations (an SOD1 mutation in six patients, a C9ORF72 mutation in five patients, and a TDP43 mutation in one patient). All patients were treated with riluzole. At the time of MRI, 10 patients (10.8%) had clinically definite ALS, 37 patients (39.8%) had clinically probable ALS, 25 patients (26.9%) had clinically probable (laboratory-supported) ALS, and 20 patients (21.5%) had clinically possible ALS according to the revised El Escorial criteria [2]. All patients were found to have clinically definite ALS during follow-up. At the time of the MRI, none of the patients had clear cognitive deficits; however, only a few patients were evaluated using a thorough neuropsychological assessment. During the follow-up, four patients were found to have developed frontotemporal dementia (one of these was a carrier of the C9ORF72 mutation). With regard to the controls, we included subjects who had undergone MRI due to experiencing headache, vertigo, or other non-specific symptoms and for whom no clear pathological changes were reported (38 with headache, 25 with vertigo, 13 with syncope, 5 with insomnia/hypersomnia, 5 with restless legs syndrome, 3 with essential tremor). The Local Ethics Committee (Comitato Etico Indipendente-AUSL Bologna, Italy) approved the study. In accordance with the Italian Guidelines for Classification and Conduction of Observational Studies established by the Italian Drug Agency “Agenzia Italiana del Farmaco—AIFA” on March 20, 2008, the requirement for informed consent from each patient was waived based on the study’s retrospective design and the use of anonymized patient data and routinely collected hospital data. The study was conducted in accordance with the Declaration of Helsinki and the principles of Good Clinical Practice (GCP).

### 2.2. MRI Data

All patients were subjected to a morphological examination using a standard 3T-MRI system (SignaEXcite, GE Healthcare, Milwaukee, WI, USA) with a standard 8-channel reel. The acquisition protocol variably included 3D-FSPGR-T1, FSE-T2, GRE-T2*, FLAIR-T2, and SWI sequences (see Table S1 for details). An average of ~30 min was required for each examination.

Two neuroradiologists (SB and LAR, who both have 15 years of experience with MRI images) examined the images to identify features of each MRI image. Each observer was blinded to the other observer’s scores, the clinical data, and the condition of the subject (patient or control).

A third rater (AFM, who has 30 years of experience with MRI images) resolved any discrepancies.

We used the method of Iwasaki [8] to identify the central sulcus and the motor cortex in MR images.

**Figure 1:**
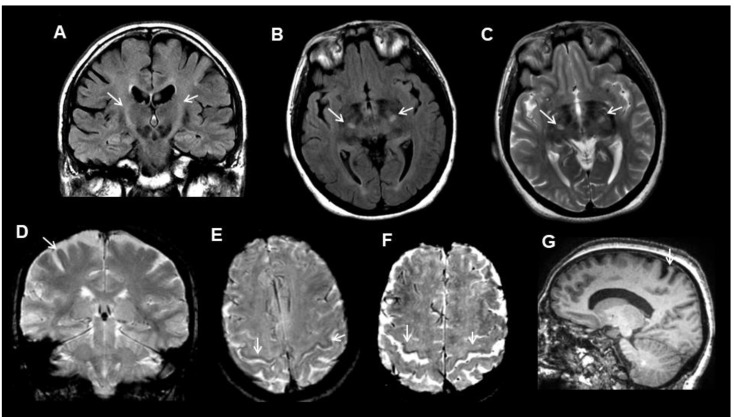
Examples of the MRI (Magnetic Resonance Imaging) features (arrows) in amyotrophic lateral sclerosis (ALS) patients. (**A**) corticospinal tract FLAIR hyperintensity in a coronal image. (**B**) corticospinal tract fluid attenuated inversion recovery (FLAIR) hyperintensity in an axial image. (**C**) corticospinal tract T2 hyperintensity in an axial image. (**D**) motor cortex T2* hypointensity in a coronal image. (**E**) motor cortex SWI hypointensity in an axial image. (**F**) motor cortex SWI hypointensity and widening of the central sulcus in an axial image. (**G**) selective motor cortex atrophy in a sagittal-T1 image.

We used transverse images at the level of the semioval center to evaluate the T2*/SWI hypointensity of the motor cortex and selective motor cortex atrophy (Figure 1).

We compared the signal intensity of the motor cortex with that of the superior frontal cortex.

The T2/FLAIR hyperintensity of the CST was evaluated in the subcortical precentral white matter and in the posterior limb of the internal capsule. The data were considered to be pathological if T2/FLAIR hyperintensity was present in both areas of interest in the MRI images (Figure 1).

The raters provided a binary “present/not present” score for each feature of interest.

### 2.3. Statistical Analyses

Statistical analyses were performed using Statistical Package for the Social Sciences (SPSS) 24.0 (IBM Corp., Armonk, NY, USA). Agreement was tested using Cohen’s k statistics. Differences between groups were evaluated using an χ2 test (*p* < 0.05; Bonferroni-corrected for multiple comparisons), followed by post-hoc two-proportion Z-test in the case of more than two groups. Sensitivity, specificity, positive predictive value (PPV), negative predictive value (NPV), and accuracy of ALS diagnosis by MRI were calculated using the clinical diagnosis as a gold standard.

The association of MRI features (i.e., CST T2 and/or FLAIR hyperintensity, MC T2* and/or SWI hypointensity, selective MC atrophy, the combination of CST T2 and/or FLAIR hyperintensity and MC T2* and/or SWI hypointensity, and the combination of CST FLAIR hyperintensity and MC SWI hypointensity) with clinical milestones (PEG/parenteral nutrition and invasive/non-invasive ventilation) were analyzed using Cox proportional hazard models after adjusting for age at MRI, sex, disease duration at time of MRI, and phenotype. The association of MRI features with death was further adjusted for any PEG/parenteral nutrition and invasive/non-invasive ventilation. As secondary analysis, we used linear regression models to test the association between the same MRI features with the decline of ALSFRS-R score from onset ((48—ALSFRS-R at MRI)/interval in months), after adjusting for age at MRI, sex, and phenotype.

## 3. Results

Demographic and clinical data on the study participants at the time of MRI are reported in Table 1. The raters’ agreement was 83–91% (kappa = 0.53–0.75, *p* < 0.001). All MRI features were significantly more prevalent in ALS patients than in controls, particularly CST FLAIR and T2 hyperintensity, which tended to be more extensive in ALS patients and even reached the brainstem and subcortical area, as well as motor cortex SWI hypointensity (Table 2).

After stratifying the patients according to phenotype at onset, all MRI features were found to be more prevalent in each ALS subgroup than in controls, with a tendency for higher prevalence in the classic and bulbar phenotypes. In particular, MC SWI hypointensity was more frequently observed in patients with the bulbar phenotype (100%), reaching significance in comparison with the prevalent LMN phenotype (Table 3).

Similar results were obtained when considering only the patients with a diagnosis of clinically possible ALS (Table S2), despite the weaker significance due to the reduced statistical power (lower sample size: 20 patients). Indeed, in this subgroup, the significance at level of the MC SWI hypointensity was lost after the Bonferroni correction (uncorrected *p* = 0.025)

No significant differences were found between mutation carriers and mutation non-carriers (Table S3), though a mild trend of mutation carriers to have less CST changes and more cortical abnormalities. Accordingly, we repeated the comparison taking out the patients with mutations (Table S4) and, subsequently, taking out the patients with mutations and those who developed dementia (Table S5). The significance at the level of MC atrophy was reduced excluding the patients who developed dementia (from *p* = 0.027 to *p* = 0.04) and disappeared excluding the patients with mutations.

In the whole group, the diagnostic accuracy was 60–72% when considering a single feature (Table 4). Among the possible combinations, the highest accuracy was obtained when considering the simultaneous presence of CST FLAIR hyperintensity and motor cortex SWI hypointensity, which mainly increased the specificity and PPV (sensitivity: 70%; specificity: 81%; PPV: 90%; NPV: 51%; accuracy: 73%) (Table 4). CST T2 hyperintensity had the highest specificity (93%) and even higher when combined with MC SWI hypointensity (97%), though low sensitivity (46%).

**Figure 2:**
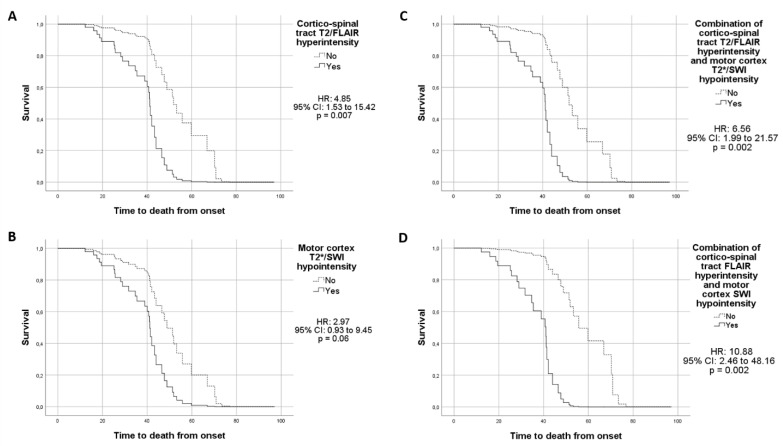
Results from the Cox proportional hazard models showing a shorter survival rate in ALS patients with CST T2/FLAIR hyperintensity (**A**), MC T2*/SWI hypointensity (**B**), combination of CST T2/FLAIR hyperintensity and MC T2*/SWI hypointensity (**C**), combination CST FLAIR hyperintensity and MC SWI hypointensity (**D**).

As regards the clinical course of the 73 patients with complete follow-up clinical information, 40 patients died (time to death 41.4 ± 20 months, mean ± SD), 28 underwent PEG/parenteral nutrition (time 35.4 ± 18.5 months, mean ± SD; 26 PEG, time 35 ± 19; 2 parenteral nutrition, time 38 ± 1), 32 underwent invasive/non-invasive ventilation (time 38.9 ± 27.9 months, mean ± SD; 30 non-invasive ventilation time 39 ± 28.6; 7 invasive ventilation time 46 ± 24.4; 5 patients underwent both). Twenty-eight patients refused invasive ventilation. The Cox proportional hazard models showed that CST T2/FLAIR hyperintensity (HR: 4.85, 95% CI: 1.53–15.42, *p* = 0.007; Figure 2A), motor cortex T2*/SWI hypointensity (HR: 2.97, 95% CI: 0.93–9.45, *p* = 0.06; Figure 2B) and, to a greater extent, the combination of these two MRI features (HR: 6.56, 95% CI: 1.99–21.57, *p* = 0.002; Figure 2C) were predictors for shorter survival. The hazard ratio was further increased by considering a combination of CST hyperintensity in only FLAIR and motor cortex hypointensity in only SWI (HR: 10.88, 95% CI: 2.46–48.16, *p* = 0.002; Figure 2D). CST T2/FLAIR hyperintensity was also associated with a shorter time to invasive or non-invasive ventilation, considering the time to reach one or the other (HR: 3.51, 95% CI: 1.35–9.12, *p* = 0.01). We found no further association between the other MRI features and the clinical milestones.

Regression models showed an association between the decline of ALSFRS-R score and the presence of CST T2 and/or FLAIR hyperintensity (B: 0.428, 95% CI: 0.013–0.843, *p* < 0.05), MC T2* and/or SWI hypointensity (B: 0.446, 95% CI: 0.051–0.841, *p* < 0.05) and the combination of CST FLAIR hyperintensity and MC SWI hypointensity (B: 0.462, 95% CI: 0.039–0.886, *p* < 0.05). We found no further association.

## 4. Discussion

In this study, we retrospectively evaluated the 3T-MRI data of a large group of ALS patients in order to identify specific changes in CST T2/FLAIR hyperintensity, MC T2*/SWI hypointensity, and selective motor cortex atrophy, which are related to UMN involvement. We found that all of these MRI features occurred more frequently in ALS patients than in controls, in line with previous results [4,5]. We obtained good diagnostic accuracy, particularly when considering CST FLAIR hyperintensity (accuracy: 72%) and motor cortex SWI hypointensity (accuracy: 71%). The combination of CST FLAIR hyperintensity and MC SWI hypointensity only slightly increased the overall accuracy compared with the single features, probably because they are highly related to each other (accuracy: 73%). However, it is interesting to note that the specificity (from 61–68% to 81%) and the PPV (from 71–83% to 90%) increased, without significantly lowering the sensitivity (from 75–76% to 70%). Interestingly, CST T2 hyperintensity had the highest specificity (93%) and even higher when combined with MC SWI hypointensity (97%); however, it had low sensitivity (46%). MRI features were more prevalent in each ALS subgroup, with a trend of higher prevalence for the classic and bulbar phenotypes, and these changes were significant when considering MC SWI hypointensity, which was present in 100% of patients with the bulbar phenotype. Unfortunately, we did not have the opportunity to include additional clinical phenotypes, such as progressive muscular atrophy, which would have been interesting to evaluate in terms of the presence of signs of UMN involvement in MRI, and which should be included in future studies. MRI features appear to be related to the evolution to dementia and to the presence of mutations, in particular with regard to the selective MC atrophy. Indeed, the significant difference of this MRI sign was reduced excluding the patients who developed dementia (only four patients), and disappeared excluding the patients with mutations. This point deserves further investigation on larger samples.

Obviously, such accuracy must be referred to the ability to detect UMN involvement as well as the clinical and neurophysiological data, rather than the ability to specifically diagnose ALS per se. Indeed, these imaging abnormalities could be present in other diseases that affect the pyramidal pathway [9,10,11]. Accordingly, for the control group, we included only subjects without a diagnosis that could be characterized by pyramidal dysfunction. However, one limitation of this study, which is due to its retrospective design, is that we did not objectively verify the absence of clinical signs of pyramidal dysfunction. We also found abnormal MRI features in some of the controls, which confirms previous results [9,10,11,12] and is why the EFNS guidelines currently do not recommend visual assessment of these alterations in brain MRIs for ALS diagnosis [9]. Notwithstanding, the CST FLAIR/T2 hyperintensity tended to be more extensive in ALS patients than in controls, with the combination of CST FLAIR hyperintensity and MC SWI hypointensity resulting in higher specificity while maintaining a good level of sensitivity, and CST T2 hyperintensity alone or in combination with MC SWI hypointensity was very specific.

The lack of the highest prevalence of the MRI features in the subgroup of UMN prevalent ALS patients does not seem to support the notion that these features reflect UMN involvement. However, we hypothesize that these signs may reflect not simply the involvement of the pyramidal pathway itself, but the rate of degeneration and/or the degree of secondary inflammation, also explaining the prognostic value that we have highlighted.

Importantly, in our cohort, ALS patients with both CST T2/FLAIR hyperintensity and MC T2*/SWI hypointensity were accurately predicted to have shorter survival, especially when considering the combination of CST FLAIR hyperintensity and MC SWI hypointensity. Furthermore, the presence of the same MRI features was associated with the decline of ALSFRS-R score.

The results of previous studies, although variable, are in line with our data. Some of these variations could be attributed to different sample sizes, magnetic field strength, sequences, image parameters, and methodological approaches. The previous studies that used conventional MRI (i.e., T2-weighted, PD-weighted, and FLAIR sequence) mainly focused on changes in signal intensity along the CST. In coronal scans, such changes are best viewed as areas of bilaterally increased signal intensity from the centrum semiovale to the brain stem [13,14]. The frequency of changes in CST signal intensity varies widely across previous studies, from 15% to 76%, depending on the sequence (T2, FLAIR, proton density) and the analyzed CST segment [9,10,14,15,16]. FLAIR imaging better detected CST signal abnormalities in ALS [15], as confirmed by our data (75% of patients with FLAIR changes vs. 46% of patients with T2 changes). CST changes probably reflect areas of Wallerian degeneration [17] at the level of the subcortical precentral white matter rather than at the level of the internal capsule [15]. This is due to the fact that CST hyperintensity in the internal capsule, probably as a result of reduced myelination, has frequently been found in healthy controls [16]. In a recent 3T-MRI study, CST hyperintensity was associated with UMN impairment and onset of the bulbar phenotype [18] and was weakly correlated with clinical scores and progression rate [18]. In a previous 1.5T-MRI study on a cohort of 24 ALS patients [19], T2-hyperintensities along the CST were more frequently detected in patients with a higher ALSFRS deterioration rate and a significantly shorter disease duration and were found to have mild predictive value for patient survival. FLAIR CST alterations exhibited stronger prognostic value for survival in a recent 1.5 T-MRI study on 28 ALS patients [20]. Our data on a larger sample and using higher magnetic field suggest an even higher prognostic value for changes in CST signal intensity, especially in combination with motor cortex hypointensity.

Motor cortex hypointensity has been increasingly evaluated in ALS patients in the last few years.

A low-signal-intensity rim, probably related to iron deposition, can be found in T2-weighted, T2*-weighted, and SWI images of the precentral cortex in ALS patients. Indeed, pathological studies examining the motor cortex of ALS patients showed iron deposition in the form of ferritin in activated microglia, specifically in the middle and deep layers [21,22]. The MRI techniques as T2, T2*, relaxometry, and SWI are especially sensitive to iron depositions [23]. A shorter T2 time in the motor cortex of some ALS patients was noted by initial studies [22,24]. However, such changes have low sensitivity and specificity as they are also observed for other neurological disorders (e.g., Alzheimer’s disease and Parkinson’s disease) and in normal aging [11]. T2*-weighted gradient echo sequences are more sensitive to the presence of iron in different tissues [23]. Data from several studies indicate that T2* shortening is histologically related to iron deposition in the microglia of the motor cortex [21,22]. Data from 7T-MR studies of the motor cortex in ALS patients also support this view [21,25]. In a follow-up study on 46 ALS patients and 26 controls, progression of the T2*-weighted hypointense area in the motor cortex was evident after 6 months and was found to be inversely correlated to disease severity [26].

SWI is a three-dimensional gradient echo sequence that enhances the contrast between substances with different magnetic susceptibilities (e.g., calcium, iron) and the surrounding background [23]. It is superior to T2 and T2*-weighted images in terms of detecting iron depositions in the motor cortex of ALS patients [27].

However, the qualitative assessment of iron-related changes in SWI was reported to have poor diagnostic accuracy. In a previous study, visual assessment of hypointensities in patients with ALS showed high sensitivity (91.15%) but low specificity (13.35%) [12]. A semi-quantitative methodology was used to reliably find iron-related hypointensities, as a marker of UMN degeneration, which were more frequently in patients with onset of the bulbar phenotype and independently of mutation status [18]. These results are confirmed by our data. Moreover, signal changes in different motor homunculus regions were associated with the site of symptom onset [18].

Globally, the reported accuracy for qualitative MRI features of ALS diagnosis varies widely across studies. This variability is probably due to differences in the used techniques and methodological approaches. The main limitation of qualitative studies, including the present study, is that they are operator-dependent. The operator-dependence could also partially account for the identification of the evaluated alterations in some controls. With regard to the relatively high prevalence of MRI features in our controls, we cannot exclude the possibility that some of these controls, who had undergone an MRI due to experiencing headache, vertigo, or other non-specific symptoms, actually presented with a pyramidal pathway pathology. This should be considered to be another limitation of our study. A further limitation of this study is the lack of an accurate cognitive status evaluation for all patients at the time of MRI, which did not allow us to evaluate the possible association of cognitive impairment with the presence of imaging abnormalities. To overcome the weaknesses of qualitative evaluation, efforts are being made to obtain reliable, standardized, operator-independent quantitative MRI parameters, such as diffusion tensor parameters or quantitative susceptibility mapping (QSM) data, and the results available to date are interesting in terms of their potential diagnostic and prognostic contribution (for more details, see [4,5,28]). However, until advanced MRI methods become easily accessible in clinical practice, the qualitative evaluation of MRI images will remain the main imaging tool in the diagnostic workup for ALS. In this scenario, our data suggest that the identification of specific MRI features, such as CST FLAIR hyperintensity and MC SWI hypointensity, using a high-field scanner in addition to the clinical and neurophysiological data may provide us with further information to assist in the clinical diagnosis of ALS. More importantly, our data indicate that such changes are predictors of shorter survival in these patients.

## Figures and Tables

**Table 1:** Characteristics of study participants.

Subjects	Tot	M/F	Age at MRI (y; Mean ± SD)	Age at Onset (y; Mean ± SD)	Disease Duration at the Time of MRI (m; Mean ± SD)	ALSFRS-R (Mean ± SD)	Phenotype at Onset			
							Prevalent UMN	Bulbar	Classic	Prevalent LMN
Patients *	93	55/38	62.8 ± 10.1	60.8 ± 10.7	25 ± 31	40.4 ± 6.2	14 (15%)	14 (15%)	47 (50.5%)	18 (19.5%)
Controls	89	56/33	60.2 ± 9.5	/	/	/	/	/	/	/
		*p* = 0.6 (χ2-test)	*p* = 0.08 (*t*-test)	/	/	/	/	/	/	/

* 12 patients had a mutation (6 had an SOD1 mutation, 5 had a C9ORF72 mutation, and 1 had a TDP43 mutation). M, male; F, female; y, years; m, months; SD = standard deviation; ALSFR-R, revised ALS Functional Rating Scale; UMN, upper motor neuron; LMN, lower motor neuron; MRI, Magnetic Resonance Imaging.

**Table 2:** Comparison of the prevalence of MRI features between ALS patients and controls.

	Corticospinal Tract FLAIR Hyperintensity	Corticospinal Tract T2 Hyperintensity	Motor Cortex SWI Hypointensity	Motor Cortex T2* Hypointensity	Selective Motor Cortex Atrophy
Patients	69/92 (75%)	42/92 (46%)	62/82 (76%)	11/43 (26%)	63/91 (69%)
Controls	28/87 (32%)	6/87 (7%)	13/33 (39%)	8/63 (13%)	43/88 (49%)
χ2-test (corrected)	*p* < 0.0001	*p* < 0.0001	*p* = 0.0011	n.s.	*p* = 0.027

Numerator indicates how many patients had the MRI feature and the denominator indicates in how many patients the sequence was available. n.s., not significant. T2* (or T2-star) is the whole name of the sequence.

**Table 3:** Comparison of the prevalence of MRI features among different ALS phenotypes.

	Corticospinal Tract FLAIR Hyperintensity	Corticospinal Tract T2 Hyperintensity	Motor Cortex SWI Hypointensity	Motor Cortex T2* Hypointensity	Selective Motor Cortex Atrophy
Prevalent UMN	9/13 (69%)	4/13 (31%)	7/12 (58%)	3/6(50%)	10/14 (71%)
Bulbar	10/14 (71%)	7/14 (50%)	10/10 (100%)	1/8 (13%)	11/14 (79%)
Classic	38/47 (81%)	27/47 (57%)	37/44 (84%)	6/19 (32%)	32/46 (70%)
Prevalent LMN	12/18(67%)	4/18 (22%)	8/16 (50%)	1/10 (10%)	10/57 (59%)
χ2-test (corrected)	n.s.	n.s.	*p* = 0.03 *	n.s.	n.s.

Numerator indicates how many patients had the MRI feature and the denominator indicates in how many patients the sequence was available. n.s., not significant. * post-hoc two-proportion Z-test (Bonferroni-adjusted) showed a significant difference between bulbar and prevalent LMN phenotypes.

**Table 4:** Diagnostic accuracy of the significant MRI features, considered individually and combined.

	Corticospinal Tract FLAIR Hyperintensity	Corticospinal Tract T2 Hyperintensity	Motor Cortex SWI Hypointensity	Selective Motor Cortex Atrophy	Both Motor Cortex SWI Hypointensity and Selective Motor Cortex Atrophy	Both Motor Cortex SWI Hypointensity and Corticospinal Tract T2 Hyperintensity	Both Corticospinal Tract FLAIR Hyperintensity and Selective Motor Cortex atrophy	Both Corticospinal Tract T2 Hyperintensity and Selective Motor Cortex Atrophy	Both Corticospinal Tract FLAIR Hyperintensity and Corticospinal Tract T2 Hyperintensity	Both Corticospinal Tract FLAIR Hyperintensity and Motor Cortex SWI Hypointensity
SENS	0.75	0.46	0.76	0.69	0.57	0.46	0.57	0.34	0.47	0.70
SPEC	0.68	0.93	0.61	0.51	0.73	0.97	0.80	0.95	0.93	0.81
PPV	0.71	0.88	0.83	0.59	0.84	0.97	0.75	0.89	0.88	0.90
NPV	0.72	0.62	0.50	0.62	0.41	0.41	0.64	0.58	0.62	0.51
ACC	0.72	0.69	0.71	0.60	0.61	0.60	0.68	0.64	0.69	0.73

SENS, sensitivity; SPEC, specificity; PPV, positive predictive value; NPV, negative predictive value; ACC, accuracy.

## Supplementary Materials

The following are available online at https://www.mdpi.com/2077-0383/9/8/2538/s1, Table S1: Details of the sequences included in the MRI protocol, Table S2: Comparison of the prevalence of MRI features between patients clinically possible ALS according to the revised El Escorial criteria and controls., Table S3: Comparison of the prevalence of MRI features between ALS patients with and without mutations, Table S4: Comparison of the prevalence of MRI features between ALS patients, excluding patients with mutations, and controls, Table S5: Comparison of the prevalence of MRI features between ALS patients, excluding patients who developed dementia, and controls.
