# Supplementary material for: Diagnostic and Prognostic Value of Conventional Brain MRI in the Clinical Work-Up of Patients with Amyotrophic Lateral Sclerosis
Source: J Clin Med. 2020 Aug 6;9(8):2538. doi: 10.3390/jcm9082538 (PMC7463468; doi:10.3390/jcm9082538)
Supplement: Supplementary file 1 [file jcm-09-02538-s001.pdf]

Supplementary Materials

Table S1. Details of the sequences included in the MRI protocol

SEQUENCES	TR (ms)	TE (ms)	FLIP ANGLE	MATRIX	THICKNESS (mm)
3D-FSPGR-T1	9	4	13°	512x512	1
FSE-T2	4000	111	90°	512x512	4
GRE-T2*	1000	20	17°	320x256	4
FLAIR-T2	9000	146	90°	320x320	4
SWI	77	42	15°	320x224	1

3D-FSPGR = Three-dimensional Fast Spoiled Gradient-Echo. FSE = Fast Spin-Echo. GRE = Gradient-Echo. FLAIR = Fluid Attenuated Inversion Recovery. SWI = Susceptibility-Weighted Imaging

Table S2. Comparison of the prevalence of MRI features between patients clinically possible ALS according to the revised El Escorial criteria and controls.

	Corticospinal tract FLAIR hyperintensity	Corticospinal tract T2 hyperintensity	Motor cortex SWI hypointensity	Motor cortex T2* hypointensity	Selective motor cortex atrophy
**Clinically possible ALS**	14/20 (70%)	8/20 (40%)	13/18 (72%)	3/10 (30%)	12/18 (67%)
**Controls**	28/87 (32%)	6/87 (7%)	13/33 (39%)	8/63 (13%)	43/88 (49%)
**χ2-test (corrected)**	$p = 0.01$	$p = 0.005$	n.s.	n.s.	n.s.

Numerator indicates how many patients had the MRI feature and the denominator indicates in how many patients the sequence was available, n.s., not significant.

Table S3. Comparison of the prevalence of MRI features between ALS patients with and without mutations.

	Corticospinal tract FLAIR hyperintensity	Corticospinal tract T2 hyperintensity	Motor cortex SWI hypointensity	Motor cortex T2* hypointensity	Selective motor cortex atrophy
**Patients without mutations**	62/80 (78%)	38/80 (48%)	54/72 (75%)	10/36 (28%)	53/79 (67%)
**Patients with mutations**	7/12 (58%)	4/12 (33%)	8/10 (80%)	1/7 (14%)	10/12 (83%)
**χ2-test (corrected)**	n.s.	n.s.	n.s.	n.s.	n.s.

Numerator indicates how many patients had the MRI feature and the denominator indicates in how many patients the sequence was available. n.s., not significant.

Table S4. Comparison of the prevalence of MRI features between ALS patients, excluding patients with mutations, and controls.

	Corticospinal tract FLAIR hyperintensity	Corticospinal tract T2 hyperintensity	Motor cortex SWI hypointensity	Motor cortex T2* hypointensity	Selective motor cortex atrophy
**Patients**	62/80 (78%)	38/80 (48%)	54/72 (75%)	10/36 (28%)	53/79 (67%)
**Controls**	28/87 (32%)	6/87 (7%)	13/33 (39%)	8/63 (13%)	43/88 (49%)
**χ2-test (corrected)**	$p < 0.0001$	$p < 0.0001$	$p = 0.0026$	n.s.	n.s.

Numerator indicates how many patients had the MRI feature and the denominator indicates in how many patients the sequence was available, n.s., not significant.

Table S5. Comparison of the prevalence of MRI features between ALS patients, excluding patients who developed dementia, and controls.

	**Corticospinal tract FLAIR hyperintensity**	**Corticospinal tract T2 hyperintensity**	**Motor cortex SWI hypointensity**	**Motor cortex T2* hypointensity**	**Selective motor cortex atrophy**
**Patients**	66/88 (75%)	41/88 (47%)	59/79 (75%)	11/41 (27%)	59/87 (68%)
**Controls**	28/87 (32%)	6/87 (7%)	13/33 (39%)	8/63 (13%)	43/88 (49%)
**χ2-test (corrected)**	$p < 0.0001$	$p < 0.0001$	$p = 0.002$	n.s.	$p = 0.04$

Numerator indicates how many patients had the MRI feature and the denominator indicates in how many patients the sequence was available, n.s., not significant.
